# Oral and intravenous iron treatment alter the gut microbiome differentially in dialysis patients

**DOI:** 10.1007/s11255-022-03377-0

**Published:** 2022-09-27

**Authors:** Huan Liu, Wenqi Wu, Yankun Luo

**Affiliations:** 1 Department of Nephrology, The Fifth Clinical Medical College of Shanxi Medical University, Taiyuan, 030000 Shanxi China; 2 Department of Thoracic Surgery, Affiliated Hospital of Beihua University, Jilin, 132000 Jilin China; 3 Department of Nephrology, Shanxi Provincial People’s Hospital, Taiyuan, 030000 Shanxi China

**Keywords:** Iron therapy, Anemia, Gut microbiota, Dialysis, CKD

## Abstract

**Objective:**

Chronic kidney disease (CKD) is often complicated by anemia, which seriously affects the quality-of-life and prognosis of patients. These patients usually need iron replacement therapy. Oral iron affects the composition and abundance of intestinal flora by increasing intestinal iron concentration.

**Methods:**

We undertook an interventional study to investigate the effects of oral versus intravenous iron therapy on the gut microbiota. Oral ferrous succinate tablets (*n = *14) or intravenous iron sucrose (*n = *14) was administered to anemic maintenance hemodialysis (MHD) patients for 2 months.

**Results:**

Oral and intravenous iron treatments had different effects on gut microbial composition and diversity. After oral iron treatment, the α-diversity was decreased, while at the phylum level, the abundance of *Firmicutes* was reduced and the abundance of *Bacteroides* was increased. At the genus level, the abundance of *Blautia* and *Coprococcus* was decreased, and the abundance of *Bacteroidetes* was increased. Oral iron therapy was associated with a higher abundance of *Lactobacillus* compared with that measured in intravenous iron-treated patients. According to metagenome function prediction analysis, oral iron increased the metabolic processes of phenylalanine, valine, leucine, and isoleucine. These changes may increase uremic toxin levels, thereby increasing the progression of renal disease.

**Conclusion:**

Iron therapy affects the diversity and composition of gut flora in MHD patients. Oral iron affects the number of bacteria and increases amino acid metabolism compared with intravenous iron. These results indicate that intravenous iron may be more appropriate for MHD patients.

**Supplementary Information:**

The online version contains supplementary material available at 10.1007/s11255-022-03377-0.

## Introduction

The human gastrointestinal (GI) tract contains over 100 trillion bacteria with the balance of the gut microbiota related closely to human health. Recently, the theory of the gut–kidney axis has become increasingly important. It has been shown that gut microbes of CKD patients are significantly altered [1], changes that may lead to a deterioration in renal function. Many non-antibiotic drugs, such as iron preparations and proton-pump inhibitors, may also cause changes in the composition of the gut microbiome [2]. In addition, the interaction between drugs and the gut microbiota may be complex and bidirectional [2].

Iron is crucial for numerous physiological and cellular processes, such as mitochondrial respiration, DNA synthesis, oxygen transport, and some metabolic pathways. MHD patients usually require oral iron preparations to supplement iron storage. However, iron supplementation may have bad effects on the gut microbiota in MHD patients, because it increases luminal iron content which will be used by some iron affinity pathogens that are detrimental to the health of patients. Some researchers have suggested that intravenous iron may have different effects on the gut microbiome than oral iron. This study in MHD patients, therefore, compared the influence of oral and intravenous iron administration on the gut microbiota and evaluated whether intravenous iron had better effects on the gut microbiome.

## Materials and methods

### Study design

This research is a non-blind randomized controlled trial which enrolled 28 patients maintained on regular HD in the Fifth Clinical Medical College of Shanxi Medical University for more than 3 months. Patients who were diagnosed with CKD5 (maintenance hemodialysis) complicated by iron deficiency (iron serum saturation <20% with or without a ferritin level <200 mg/L) were included in the study. The age inclusion criteria were >18 years and <75 years, while the exclusion criteria were blood transfusions, antibiotic usage, diarrhea, the combination of folate or vitamin B12 deficiency in the last 3 months, gastric ulcer, gastric hemorrhage, additional probiotics, hormone use, pre-existing hematological disease, and cancer. Recruited patients were randomized to receive either oral iron (200mg of ferrous succinate tablet, once a day) or intravenous iron (100 mg of iron sucrose, 3 times a week), which was used in previous trials [3, 4] and according to KDIGO clinical practice guideline for anemia in chronic kidney disease. Oral iron treatments were continued for at least 2 months [3, 4]. Intravenous iron was infused at least ten times. Follow-up iron treatment depends upon hemoglobin and ferritin levels.

### Sample collection, DNA extraction, and 16S rRNA gene amplicon sequencing

Stool specimens were collected at baseline and 2 months after iron treatment. A stool collection kit and a pamphlet with sampling instructions were given to each participant. The stool samples were collected in the morning, frozen within 30 min, and then stored at − 80 °C until sequencing was completed. The OMEGA Soil DNA Kit (D5625-01) (Omega Bio-Tek, Norcross, GA, USA) was used to extract total community genomic DNA from 50 mg of feces. Cleavage and digest of the DNA were carried out in a lysis solution containing protease and the DNA and then adsorbed in a binding solution containing magnetic particles. The purified DNA was used for 16SrRNA gene sequencing using primers targeted to the V3–V4 region (338F(5’-barcode + ACTCCTACGGGAGGCAGCA-3’) and (806R (5’-GGACTACHVGGGTWTCTAAT-3’)). The 16S rRNA genes were amplified in technical triplicates. The Illumina NovaSeq (NovaSeq 6000 SP reagent kit (500cycles)) was used to perform paired-end sequencing (2 × 250 base pairs) in a single batch.

### Statistical Analysis

The QIIME 2 microbial analysis platform was used to analyze and map the microbial data. The survival feature sequence ASVs were denoised using DADA2. The Mann–Whitney U test was used to compare variance in the Chao1, observed species, Shannon diversity index, Simpson diversity index, Faith's phylogenetic diversity, Pielou's evenness, and Good’s coverage in intra-sample comparisons (α-diversity). Intergroup comparisons (β-diversity) were assessed using principal coordinate analysis (PCoA). The PCoA analysis was performed by calculating Jaccard and Bray–Curtis distance matrices [5]. The α-diversity index and PCoA diagram were drawn using the R software package “ggplot2”. LEfSe analysis was used to identify different species between the two treatment groups. The LDA effect was calculated and the group with LDA score >2 and *P* < 0.05 was considered statistically significant [6]. We used PICRUSt2 to predict the metagenomes. The metagenome was then divided into three layers and abundance clustering performed on the functions of multiple samples according to the KEGG database annotation that was then used to construct a pathway clustering heat map. Differences in results between the two groups with *P* < 0.05 were considered statistically significant (Supplementary Material 1).

## Results

**Fig. 1:**
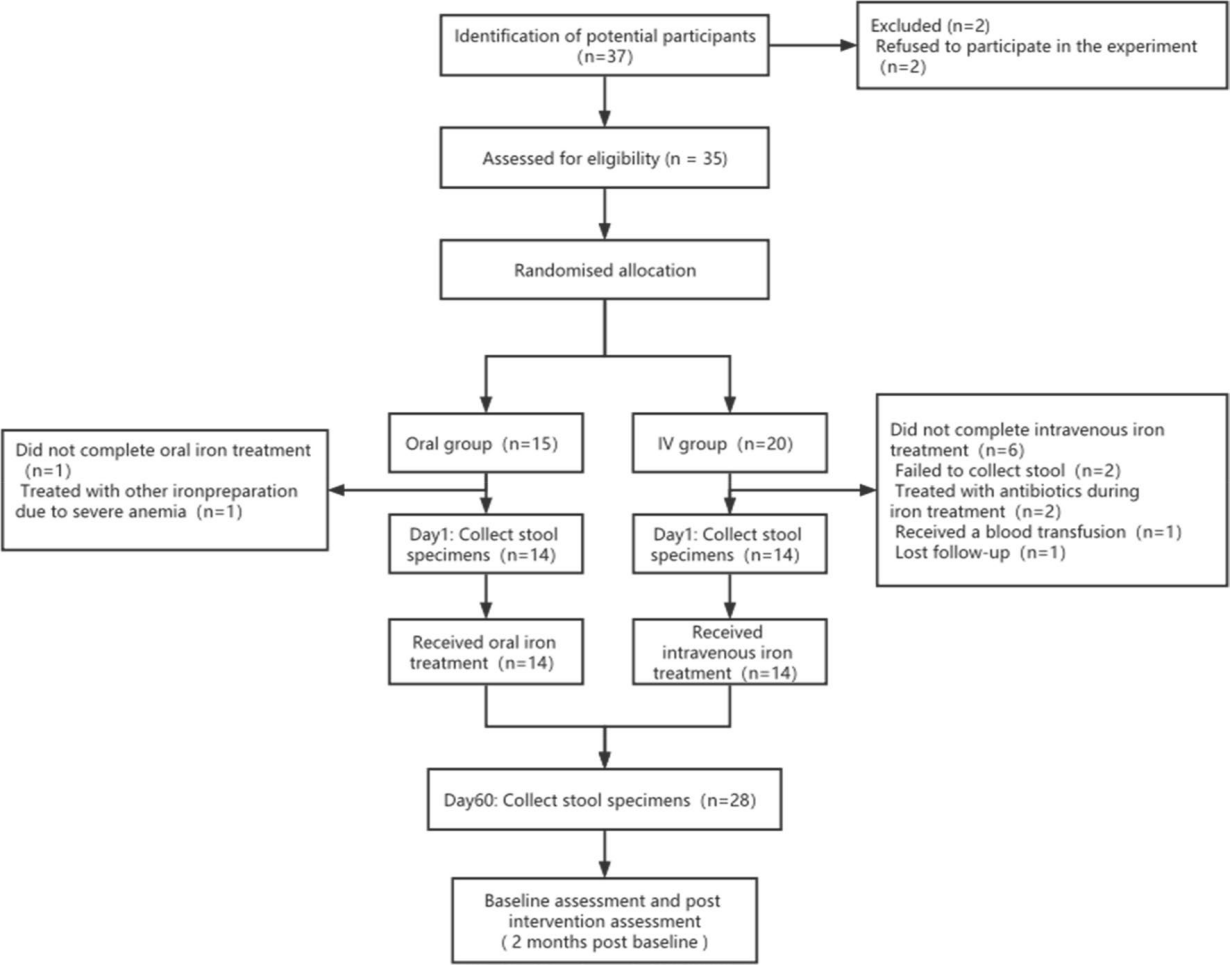
Participants’ flowchart

A total of 28 MHD patients (10 females, 18 males) were enrolled in the study, with 14 receiving oral iron and 14 receiving intravenous iron (Fig. 1). As shown in Table 1, the oral and intravenous groups were matched for age, gender, BMI, serum ferritin, and mean iron saturation level.

**Table 1:** Comparison of the clinical characteristics of the two treatment groups

Patient characteristics	Oral iron (*n = *14)	Intravenous iron (*n = *14)	*P* value
Age (years)	54.21 ± 16.76	59.36 ± 12.33	0.330
Female/male, *n* (%)	5 (36)/9 (64)	5 (36)/9 (64)	1.000
BMI (kg/m^2^)	23.55 ± 3.67	21.69 ± 2.83	0.923
Inclusion Hb (g/L)	90.57 ± 14.22	83.86 ± 8.74	0.144
Serum ferritin (ng/mL)	29.60 (56.70)	20.70 (55.33)	0.610
Transferrin saturation, %	14.87 ± 6.00	14.04 ± 5.89	0.715
Diabetes, *n* (%)	9 (64)	8 (57)	0.550
Hemodialysis mode	HD	HD	–
Frequency of hemodialysis therapy (times/week)	3/w	3/w	–

The continuous variables are expressed as means ± standard deviations, while categorical variables are expressed as mean percentages. BMI, body mass index. Hb, Hemoglobin. HD, hemodialysis.

### Bacterial diversity following iron therapy

**Fig. 2:**
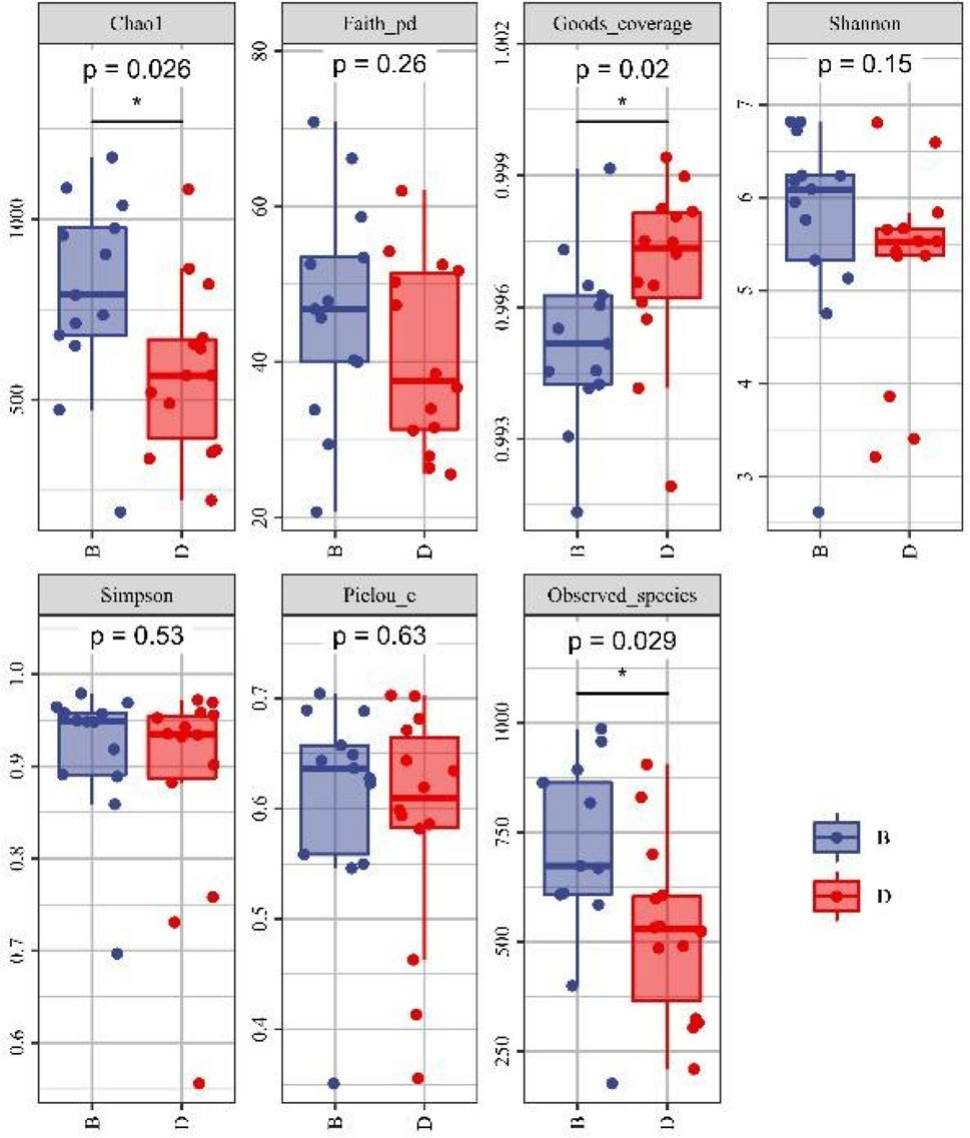
The α-diversity of the oral iron group was altered following iron therapy. Estimation of α-diversity using Chao1, Faith’s phylogenetic diversity, Goods coverage, Shannon diversity, Simpson diversity, Pielou’s evenness, and observed species was considerably lower in the oral group than in the intravenous group. B, after oral iron therapy and D, after intravenous iron therapy. *P < 0.05

A rarefaction curve was constructed to evaluate the depth of OTU clustering. The data showed that the curves of each sample tended to be flat, indicating that the current sequencing depth reflected the diversity of intestinal flora (supplemental materials 2). Species accumulation curves were used to estimate whether the sample size is sufficient. The curve keeps an upward trend, which indicates that the sample size is not sufficient (supplemental materials 3). A comparison of the α-diversity statistics showed that patients treated with oral iron had less Chao1 and Observed species compared to the IV group (Fig. 2, *P* < 0.05). The Shannon and Simpson diversity indices were similar between the two groups. The Chao1 and Observed species are established indicators of species richness, and therefore, our data indicate that oral iron therapy reduces the abundance of bacterial richness without affecting diversity.

**Fig. 3:**
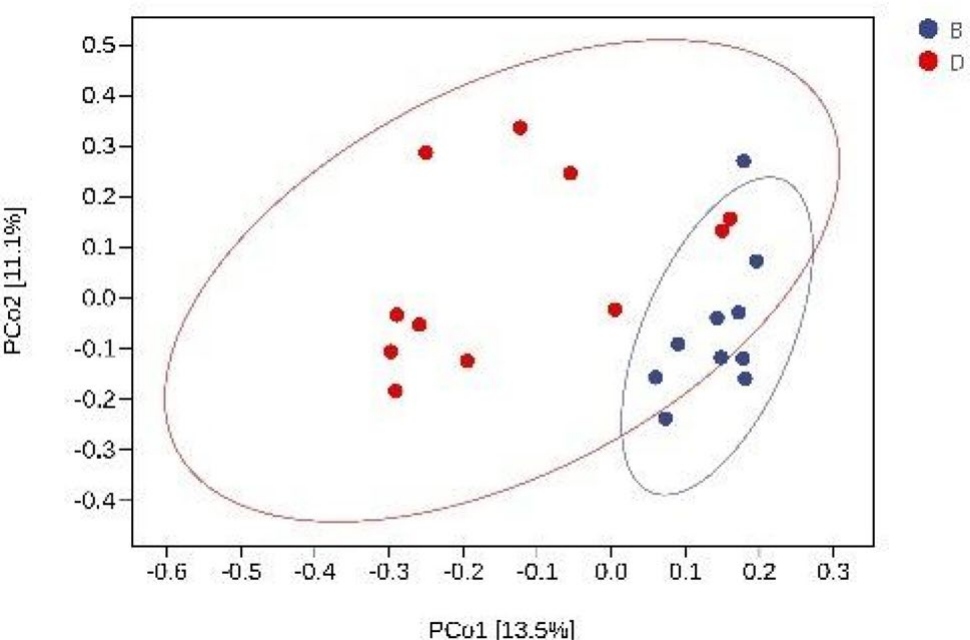
Principal coordinate analysis (PCoA) plots were constructed based on Bray–Curtis distance matrix. β-Diversity showed obvious bacterial community clusters between the oral and intravenous groups (*P* < 0.05). B, after oral iron therapy and D, after intravenous iron therapy

Principle coordinate analysis (PCoA) was used to examine differences in the fecal metabolomes between the oral and IV groups. Figure 3 shows that oral administration of iron resulted in different gut microbiome clusters compared to those formed with intravenous iron (Fig. 3, *P* < 0.05). This suggests that oral and intravenous iron therapy has different effects on bacterial diversity, with oral iron therapy reducing bacterial richness. The β-diversity of the MHD patients also differed with oral and intravenous iron treatment.

**Fig. 4:**
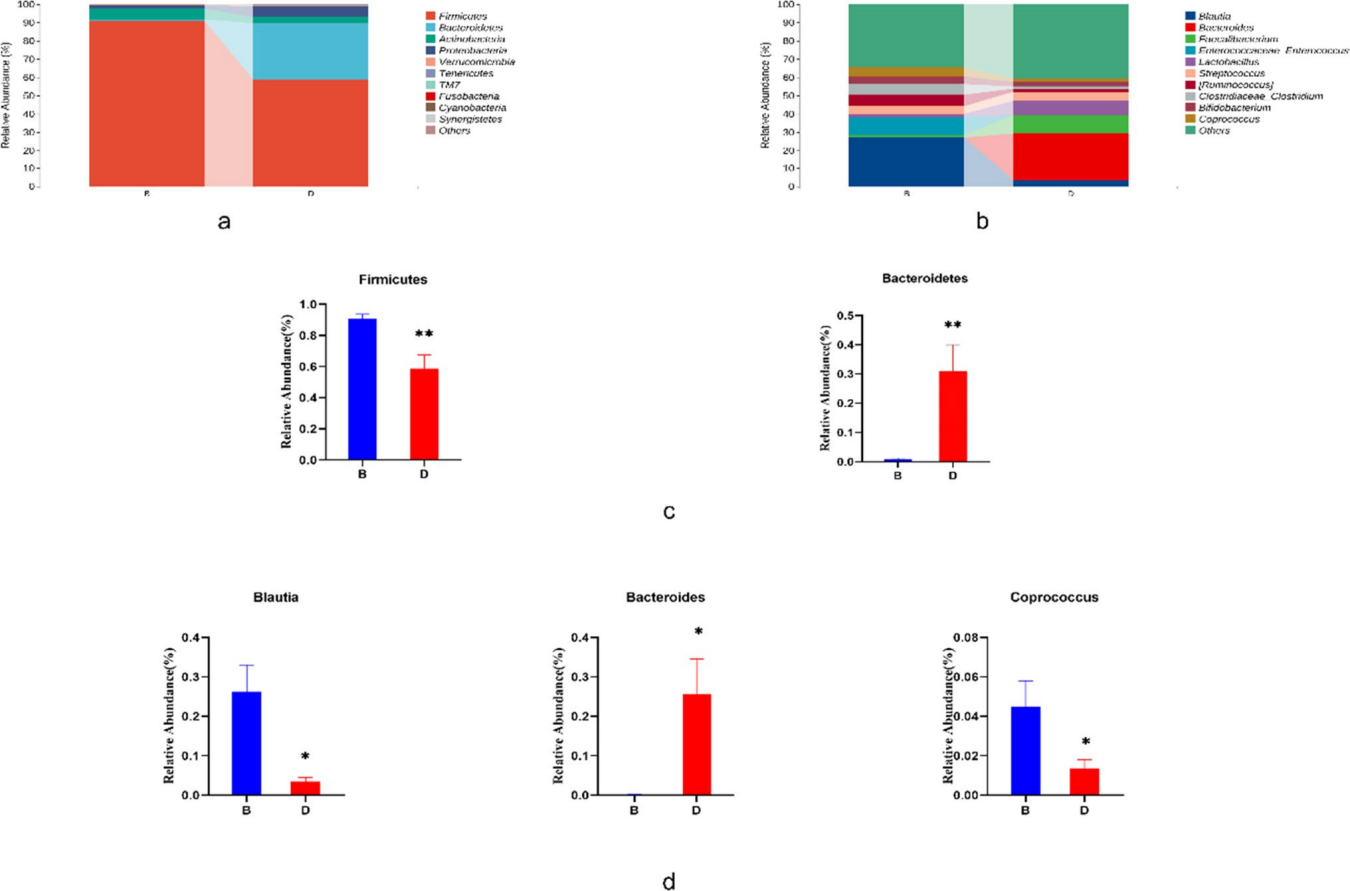
Average bacterial community compositions at the phylum **A** and genus **B** levels in both groups. Relative abundance of dominant bacterial taxa at the phylum **C** and genus **D** levels with significant differences between the two treatment groups. *P < 0.05, **P < 0.01. B, after oral iron therapy and D, after intravenous iron therapy

We examined the composition of the gut microbiota in the MHD patients from phylum to genus level and also analyzed the data to identify significant differences in taxonomic composition between the oral and intravenous groups (Fig. 4A and B). *Firmicutes* were the dominant phylum in MHD patients. Compared to intravenous iron therapy, oral iron therapy decreased the relative abundance of *Firmicutes* and significantly increased the abundance of *Bacteroidetes* at the phylum level (*P* < 0.01, Fig. 4C). At the genus level, the abundance of the *Blautia* and *Coprococcus* was significantly decreased, while the abundance of *Bacteroidetes* was increased significantly in the oral group compared to that measured in the intravenous group (*P* < 0.05, Fig. 4D).

### Bacterial communities in oral and intravenous iron-treated patients differ

**Fig. 5:**
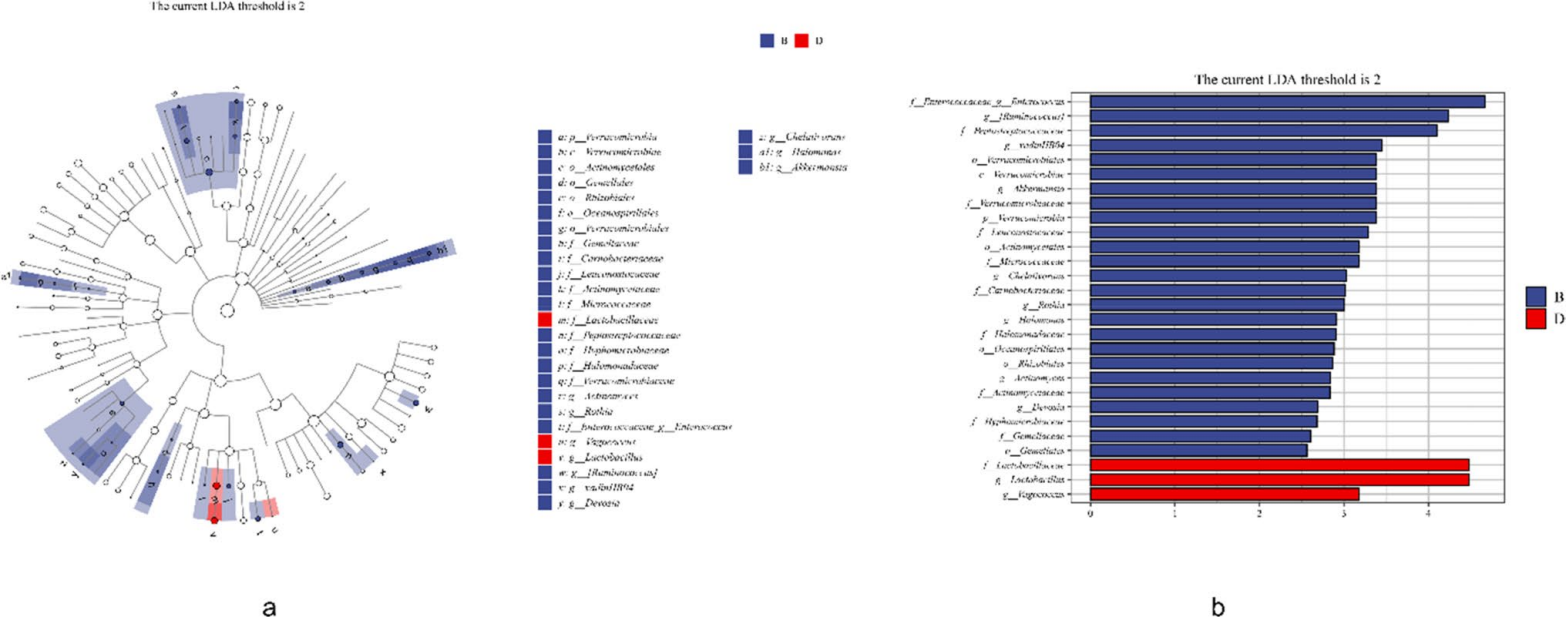
LEfSe to identify bacterial taxa that shows significant differences in enrichment between the two treatment groups. The abundance of the taxa from phylum to genus levels is included. **A** Taxonomic cladogram of the LEfSe analysis; **B** histogram of the linear discriminant analysis scores; B, after oral iron therapy and D, after intravenous iron therapy. A LDA > 2 and *P* < 0.05 were considered statistically significant

We use LEfSe to determine differences in the bacterial taxa between oral and intravenous iron treatment. The microbiota of the IV group showed a greater abundance of the *Verrucomicrobiae* class, *Verrucomicrobiales*, *Actinomycetales*, *Oceanospirillales*, *Rhizobiales*, and *Gemellales* orders, *Enterococcaceae*, *Peptostreptococcaceae*, *Verrucomicrobiaceae*, *Leuconostoaceae*, *Micrococcaceae*, *Camobateriaceae*, *Halomonadaceae*, *Actinomycetaceae*, *Hyphomicrobiaceae*, *and Gemellaceae* families, and *Enterococcus, Ruminococcus*, *VadinHB04*, *Akkermansia*, *Chelativorans*, *Rothia*, *Halomonas*, *Actinomyces*, and *Devosia* genus. The *Lactobacillaceae* family, and the *lactobacillus* and *vagococcus* species were found in greater abundance in the oral group (*P* < 0.01, Fig. 5). These results suggest that the gut microbiome in MHD patients responds differently to oral and intravenous iron therapy.

### Metagenomes function prediction analysis

**Fig. 6:**
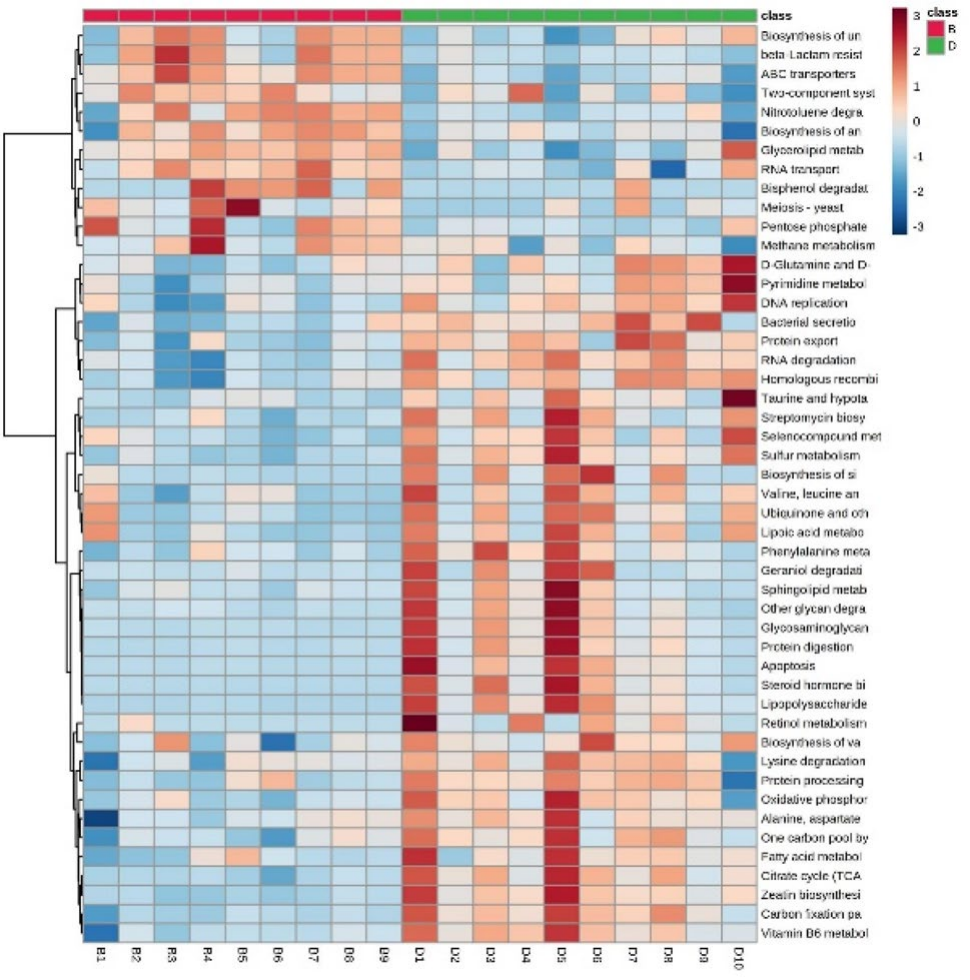
Metabolite pathway heatmap for metagenomic prediction between the oral and IV groups. *P* < 0.05 were considered statistically significant. B, after oral iron therapy and D, after intravenous iron therapy

We used PICRUSt2 software to perform a metagenomic prediction analysis on the 16S rRNA data. This analysis showed 48 gene categories were significantly regulated. The oral group had 36 upregulated genes and 12 downregulated genes when compared to the IV group. Mapping the gene family to KEGG databases was used to obtain abundance data of each metabolic pathway in the samples. By exploring the KEGG pathway or module, we found that amino acid metabolism-related genes increased in the oral group, such as those involved in phenylalanine, alanine, aspartate, and glutamate metabolism, and valine, leucine, and isoleucine degradation. The IV group was associated with an increased abundance of genes for ABC transporters, degradation of nitrotoluene, glycosaminoglycan, and bisphenol, metabolism of glycerolipid and methane, resistance to beta-lactam, biosynthesis of ansamycins and unsaturated fatty acids, the two-component system, yeast meiosis, and the pentose phosphate pathway (Fig. 6).

## Discussion

Anemia is a common complication in CKD patients, especially in those with advanced CKD. Anemia usually results from absolute iron deficiency or a functional deficiency [7]. People with these abnormalities can choose oral or IV iron for treatment. There are many GI side effects caused by oral iron, such as nausea, vomiting, upper abdominal pain, diarrhea, and constipation [7, 8]. Excess luminal iron can induce oxidative stress and lipid peroxidation which damages intestinal epithelial cells, resulting in destruction of the mechanical barrier [9]. In addition, oral iron can change the composition of the gut microbiota and gut metabolome, which may have adverse effects on kidney function by producing more uremic toxins [3, 10]. Therefore, we collected fecal samples of MHD patients to analyze the influence of oral (PO) and intravenous (IV) iron therapy on the gut microbiota by comparing bacterial diversity and composition in the two groups.

In recent years, an increasing number of studies have reported an association between the gut microbiome and CKD. The composition and diversity of the gut microbiome in CKD patients is changed significantly, with studies showing that compared to healthy controls, the relative abundance of 190 bacterial operational taxonomic units (OTUs) was altered, and bacterial diversity was reduced [11, 12]. Plasma trimethylamine-N-oxide (TMAO) levels were also elevated due to dysbiosis of the gut microbiome, which would be harmful to the long-term survival of CKD patients [13, 14]. Several recent studies have confirmed that iron supplementation has an effect on the intestinal flora of living animals, increasing the abundance of other pathogens, such as *Escherichia coli* and *Streptococcus*, and decreasing the prevalence of *Bifidobacterium* and *Lactobacillus* [15, 16], changes that may have adverse effects on kidney function.

Our study compared the influence of different irons on the gut microbiome using 16sRNA sequencing. We found that oral and intravenous iron supplements had different effects on gut microbiota diversity, bacterial taxa, and predictive metagenomics in patients with MHD. These findings were consistent with those of the following earlier studies. Similar results were found by Lee [3] in patients with inflammatory bowel disease (IBD) who showed that oral and IV iron therapies differentially affected bacterial communities. The presence of particular molecular bacterial species was changed by oral iron treatment, which resulted in elevation of luminal iron concentrations. Based on these results, IV iron therapy was considered to have beneficial effects on the gut microbiota [3]. Our study also found that bacterial diversity and composition changed markedly after iron therapy. The microbial diversity of the oral group was significantly different from that of the IV group, while the microbial richness of the oral group was significantly reduced compared to that measured in the IV group. This suggested that IV iron therapy may be a healthier option [17] and result in a more diverse microbiota compared with that achieved by oral iron therapy. There is also evidence that gut microbial diversity is decreased significantly in patients with CKD compared to that in healthy controls [11]. These results support the possibility that oral administration of iron may have adverse effects in MHD patients.

In the oral group, the abundance of the phylum *Firmicutes* was decreased significantly, while *Bacteroidetes* was increased significantly. This resulted in a reduction in the *Firmicutes*/*Bacteroidetes* (F/B) ratio. It has been reported that obesity is associated with an increased F/B ratio, while IBD is associated with a decreased F/B ratio, with the genus *Lactobacillus* being the most studied probiotic for adjusting the ratio [18]. However, opinions differ on these associations, and it is currently difficult to relate the F/B ratio to a specific health condition and even obesity [19]. At the genus level, the abundance of *Bacteroides* was increased significantly in the oral group in our study. Similarly, it has been reported that mice fed different concentrations of iron had an increased abundance of *Bacteroides* [20]. A previous study in humans showed that *Bacteroides* played a complex role, with some subclasses of *Bacteroides* having beneficial humancial effects, while others were harmful [21]. There is also evidence that *Bacteroides dorei* and *Bacteroides vulgatus* reduce the production of lipopolysaccharides by gut microbiota and alleviate endotoxemia, changes which are both beneficial for preventing atherosclerosis [22].

In contrast, we found short-chain fatty acid (SCFA) producing bacteria, including species of *Coprococcus*. *Blautia*, and *Lachnospiraceae*, were less abundant in the oral group. SCFAs are the main bacterial metabolites produced by specific anaerobic bacteria that ferment non-starch polysaccharides (NSP) and resistant starch (RS) in the colon. This mainly includes acetic acid, propionic acid, and butyric acid. SCFAs are signaling molecules that mediate interactions between the diet, gut microbiota, and the host, and play an important role in immunity, metabolism, and the endocrine system [23]. SCFAs also have an anti-inflammatory function by regulating immune cell chemotaxis, the release of reactive oxygen species (ROS) and cytokines, and there is also evidence that they help preserve gut health by preventing and improving a range of ailments, including cancer [24].

Butyrate is an SCFA that is regarded as a source of colon energy. Butyrate can greatly improve intestinal barrier function while also reducing the inflammatory response. In addition, butyrate maintains intestinal immune balance and has a potential protective effect against IBD pathogenesis [25]. Interestingly, a recent study conducted in mice with diabetic nephropathy showed that butyrate can reduce skeletal muscle atrophy in diabetic nephropathy by stimulating the FFA2-mediated PI3K/AKT/mTOR signaling pathway [26]. A decrease in *Coprococcus* also has adverse effects on health. A recent study reported that *Coprococcus* plays an important role in health by producing vitamin B and SCFAs [27]. A systematic review showed that *Coprococcus* was decreased in patients with CKD and led to a decrease in butyrate [28], which is known to protect kidneys by GPR109a- and epigenetic-mediated mechanisms [29]. A previous study also showed an increase in the abundance of *Lachnospiraceae* in the IV iron group [30], which was reduced in patients with CKD [28]. Earlier research reported that *Blautia* was reduced significantly in people with CKD [11]. However, another study suggested that *Blautia* may be a new functional genus with potential probiotic properties [31], a finding inconsistent with previous research results. It is reported that *Blautia* is positively correlated with SCr and BUN [11]. These results indicate that a decrease in SCFA-producing bacteria in gut flora associated with oral administration of iron is damaging to the health of CKD patients.

Our study showed that there were differences in two bacterial taxa between the oral and IV groups. This result was similar to that reported in a previous study that showed IBD patients who developed different microbial taxa after receiving various iron therapies [3]. According to our results, the abundance of *Lactobacillus* was higher in the oral group compared to that measured in the IV group. *Lactobacillus* is one of the probiotics, and has been shown to correlate negatively with SCr and BUN levels [11]. When compared to intravenous iron treatment, Phipps et al. demonstrated that the abundance of *Lactobacillus* was increased after oral iron treatment in colorectal cancer patients [4]. Taken together, these results support our conclusion that patients receiving oral and intravenous iron treatment had diverse bacterial communities. Functional potential analysis using PICRUSt2 showed that oral iron supplementation increased the metabolic processes of phenylalanine, valine, leucine, and isoleucine. Amino acids are the precursors of uremic toxins. The spectrum of intestinal amino acid metabolism becomes disordered as CKD progresses, a change which is linked to intestinal microbiota dysbiosis and metagenomic alterations [32]. In addition, protein and amino acid metabolism produces precursors of uremic toxins, such as indole and p-cresol, which are further metabolized to indoxyl sulfate (IS) and p-cresyl sulfate (pCS) in the intestine and liver [33]. IS induces fibrosis-, inflammatory-, and obesity-promoting effects through the nuclear factor-Κb (NF-κB) and mitogen-activated protein kinase (MAPK) pathways [34]. IS also decreases the viability of endothelial and vascular smooth muscle cells, thereby increasing the risk of cardiovascular disease [35, 36]. PCS causes cardiomyocyte apoptosis and diastolic dysfunction through activation of NADPH oxidase and production of ROS [36]. Because iron affects amino acid metabolism, CKD patients are at a disadvantage. In the future, regulating intestinal amino acid metabolism could be a potential technique to slow the progression of CKD.

## Conclusions

In conclusion, we analyzed the effects of iron administration on intestinal flora in maintenance hemodialysis patients and compared the impacts of ferrous succinate and iron sucrose. The findings showed that the species composition and diversity of gut microflora changed significantly after iron therapy. Oral iron reduced alpha diversity and the abundance of SCFAs-producing bacteria in patients with MHD when compared to the changes caused by intravenous iron. However, the *Lactobacillus* genus was more abundant in patients on oral iron therapy, which is beneficial for patients with MHD. However, it is necessary to validate this finding in vitro. PICRUSt2 function prediction analysis showed that oral iron increased amino acid metabolism, which may increase the progression of renal disease. This could be linked to cardiovascular disease and all-cause mortality. As a result, oral iron therapy may be more hazardous to intestinal bacteria, while intravenous iron therapy may be more appropriate for patients with renal anemia. The current study had several limitations in that various factors such as nutrition may interfere with intestinal flora, and also, the sample size was relatively small. Furthermore, we only observed changes in flora over the short term and it is possible that different intervention times may provide different microbiological findings. Further metabolomics analysis is therefore required to find a clinical correlation between different microbial communities after iron therapy and all-cause mortality and cardiovascular disease risk in patients with MHD.

## Supplementary Information

Below is the link to the electronic supplementary material.

Supplementary file1 (DOCX 217 kb)

Supplementary file2 (DOCX 80 kb)

Supplementary file3 (DOCX 17 kb)

## Data Availability

All data in this study can be obtained with the permission of the author.
