# Supplementary figures and images for: Oral and intravenous iron treatment alter the gut microbiome differentially in dialysis patients
Source: Int Urol Nephrol. 2022 Sep 27;55(3):759–67. doi: 10.1007/s11255-022-03377-0 (PMC9957911; doi:10.1007/s11255-022-03377-0)

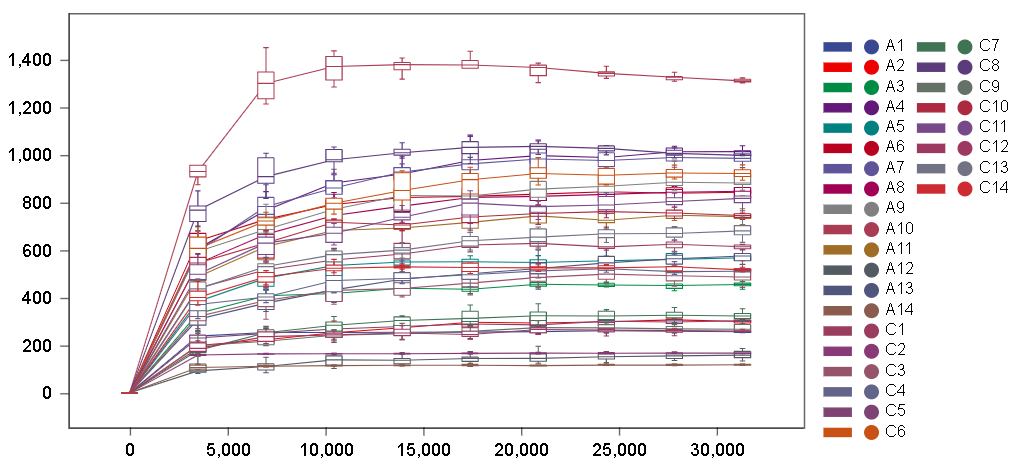

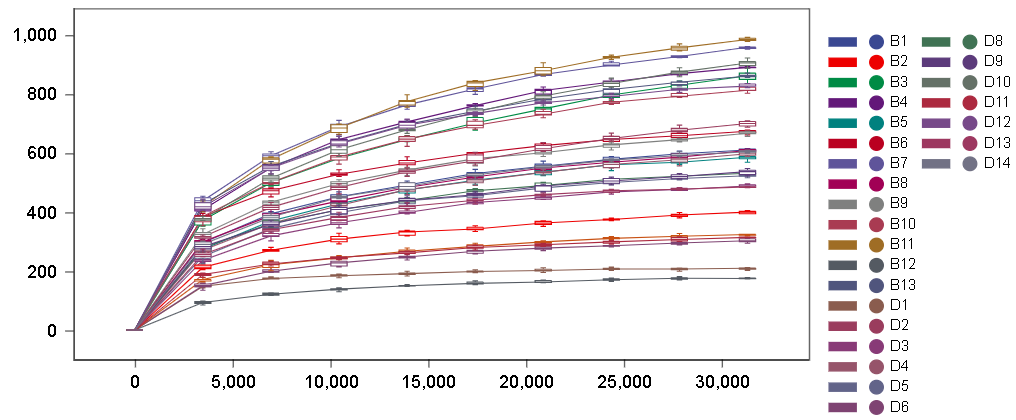
**Supplementary material 2**

**Rarefaction curve**

Supplement: Supplementary file 1 — Supplementary file1 (DOCX 217 kb) [file 11255_2022_3377_MOESM1_ESM.docx]

**Supplementary material 3**

**
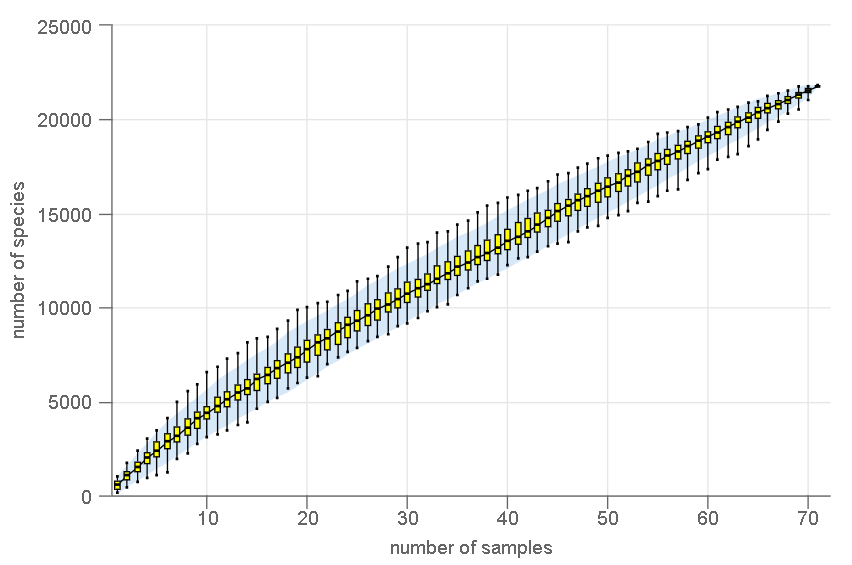
**


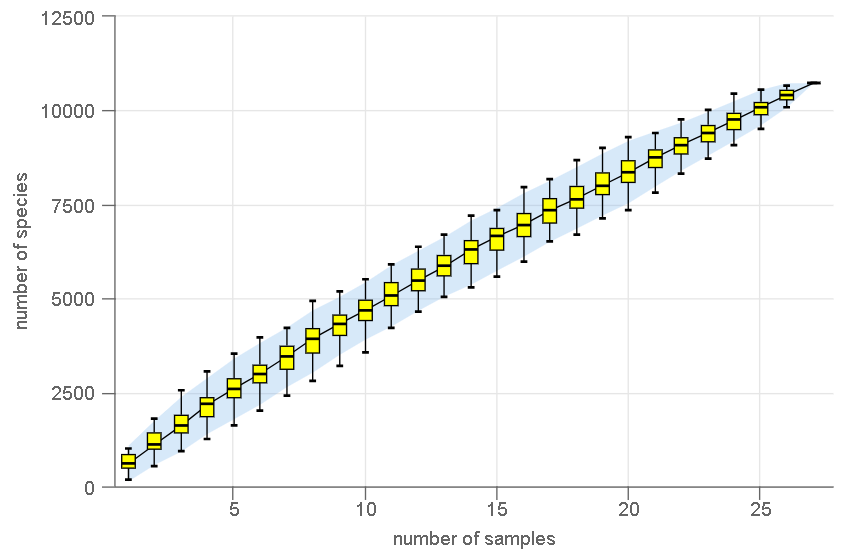
**Species accumulation curves**

Supplement: Supplementary file 2 — Supplementary file2 (DOCX 80 kb) [file 11255_2022_3377_MOESM2_ESM.docx]
