# Supplementary material for: Oral and intravenous iron treatment alter the gut microbiome differentially in dialysis patients
Source: Int Urol Nephrol. 2022 Sep 27;55(3):759–67. doi: 10.1007/s11255-022-03377-0 (PMC9957911; doi:10.1007/s11255-022-03377-0)
Supplement: Supplementary file 3 — Supplementary file3 (DOCX 17 kb) [file 11255_2022_3377_MOESM3_ESM.docx]

**Supplementary Material 1**

| Method | Meaning |
| --- | --- |
| Alpha diversity analysis | An index of species richness, diversity, and evenness in the local homogeneous habitat, also known as habitat diversity. |
| Beta diversity analysis | A diversity index focuses on the comparison of diversity between different habitats, that is, the differences between samples. |
| LEfSe analysis | The goal of the LEfSe analysis was to find robust differences between groups, known as marker species. |
| Function prediction analysis | A method to predict the functional abundance of a sample through the abundance of the sequence of the marker genes in the sample. |
